# *cep-1/p53*-dependent dysplastic pathology of the aging *C. elegans* gonad

**DOI:** 10.18632/aging.100448

**Published:** 2012-04-30

**Authors:** Mathew D. McGee, Nicholas Day, Jill Graham, Simon Melov

**Affiliations:** Buck Institute for Research on Aging, Novato, CA 94945 USA

**Keywords:** aging, p53, pathology, C. elegans, gonad

## Abstract

The *C. elegans* germline and somatic gonad are actively developing until the animal reaches adulthood, and then continue to undergo striking changes as the animal ages. Reported changes include a depletion of available sperm, a decrease in oocyte quality up till mid-life, a reduction in germline nuclei, a decrease in fertility, and an accumulation of DNA in the midbody of aging *C. elegans*. Here, we have focused on the aging gonad in old animals, and show in detail that the aging gonad undergoes a massive uterine growth composed of endoreduplicating oocytes, yolk, and expanses of chromatin. We use a novel series of imaging techniques in combination with histological methodology for reconstructing aged worms in 3-dimensions, and show in old animals growing masses swelling inside the uterus to occupy most of the diameter of the worm. We link this accelerated growth to the *cep-1/p53* tumor suppressor. Because *cep-1* is required for DNA damage induced apoptosis, and *daf-2* limits longevity, these results suggest a role for age-related DNA damage in dysplastic uterine growths, which in some respects resemble premalignant changes that can occur in aging mammals.

## INTRODUCTION

Much of the pathobiology of aging *C. elegans* and the aging germline remains relatively poorly described despite the widespread use of *C. elegans* as a model system to study aging. The aging *C. elegans* germline has been reported to undergo a series of changes up to midlife [1,2]. After approximately 8 days of age, the pool of sperm that are produced during larval development are depleted and few viable embryos are produced despite a continuous supply of oocytes. In the absence of sperm, the buildup of RNP granules is thought to facilitate cell cycle arrest in unfertilized oocytes for up to several days [3,4]. Eventually, oocytes bypass the prophase I diakinesis arrest [5] but fail to fully complete anaphase I [6]. Because they lack the sperm-contributed centrioles required for cytokinesis [7,8], these unfertilized oocytes would likely undergo endoreduplication [9,10] as worms age, rather than mitosis.

In mid-life (approximately 8-12 days of age), concurrent with sperm depletion, is a decrease in oocyte quality that also prevents the development of viable embryos even in the presence of viable sperm [11,12]. This decrease in oocyte quality with age functions at least partially through the insulin signaling and TGF-β pathways from the somatic gonad. Low quality oocytes can have various defects including small size, apparent cavities, increased aneuploidy, or cluster together in the uterus [11,13]. The apoptotic pathway is also required to maintain oocyte quality, as a loss of apoptosis in the germline causes an early loss of reproductive capacity and an earlier incidence of abnormal oocytes [14,15]. This decline in oocyte quality is also accompanied by a substantive increase in genome copy number, which is due to proliferation of the genome in the germline with age [16].

Endoreduplication has been well described in young animals, but not in worms older than 11 days of age or so (middle aged worms). Animals with a substantive amount of endoreduplication result in what is generally called the Emo (endomitotic) phenotype. This effect was first described in animals that were depleted of sperm [10,17]. Subsequently, the phenotype was observed in a Sec61p protein translocation mutant that causes defective ovulation [9]. Several other genes that affect ovulation can also cause an Emo phenotype [5], and is generally caused by inappropriate maturation of unfertilized oocytes. Therefore, many mutants or treatments that prevent fertilization could potentially cause an Emo phenotype. In an aging context, this has been described to some degree in a previous report from our laboratory [16], and more recently observed in oocytes from 8 day old animals [11].

Using imaging techniques in combination with a novel histological methodology for reconstructing aged worms in 3-dimensions, we characterize in detail here for the first time the development of large uterine masses in aging *C. elegans* which arise from unfertilized oocytes that fail to be expelled from the vulva. Although we and others have commented on early stages of this phenotype [11,16], we report here a more detailed analysis of the progression of the massive age-related uterine growths that swell the uterus and fill most of the diameter of the worm. This advanced age germline phenotype causes other internal organ systems, such as the intestine, to become compressed, which likely has multiple deleterious functional outcomes in worms of advanced age. We observe a high degree of individual variation in the aging germline phenotype, despite animals being raised in identical conditions with an identical genetic background. We also report here for the first time a detailed description of an advanced age Emo phenotype, with uterine masses appearing to be primarily a combination of endoreduplicating oocytes, clusters of cells and/or nuclei, masses of chromatin, and extracellular yolk protein. We report a retardation of age-related uterine growths in the *daf-2* insulin-signaling mutant, consistent with prior studies showing reduced germline tumor growth [18] and reduced endomitotic phenotype at older ages [11,16]. Perhaps more significantly, we report that the tumor suppressor *cep-1*/p53 mutant has a more severe endomitotic phenotype at younger ages than wild-type animals. We also mined a pre-existing whole-genome expression profiling study of aging in *C. elegans* [19] to show that the transcriptional abundance of *cep-1*/p53 statistically significantly declines with age. *Cep-1*/p53 is known to be involved in DNA damage-induced apoptosis in *C. elegans* [20,21] and there has been some evidence of impaired DNA damage response in older animals [22]. Our data therefore provides evidence for a pathological role for DNA damage in old animals. However, p53 has also been shown to be a regulator of growth via the IGF-1/mTOR pathway [23], and is an important part of the response to genotoxic stress [24,25]. Hence, it is likely that reducing levels of *cep-1*/p53 with age, results in a complex phenotype including a massive dysplastic growth in the uterus, concomitant with a profound increase in genome copy number, substantially higher than that seen in young *C. elegans* at the peak of reproductive potential.

## RESULTS

### Wild-type *C. elegans* accumulate large uterine growths with age

**Figure 1:**
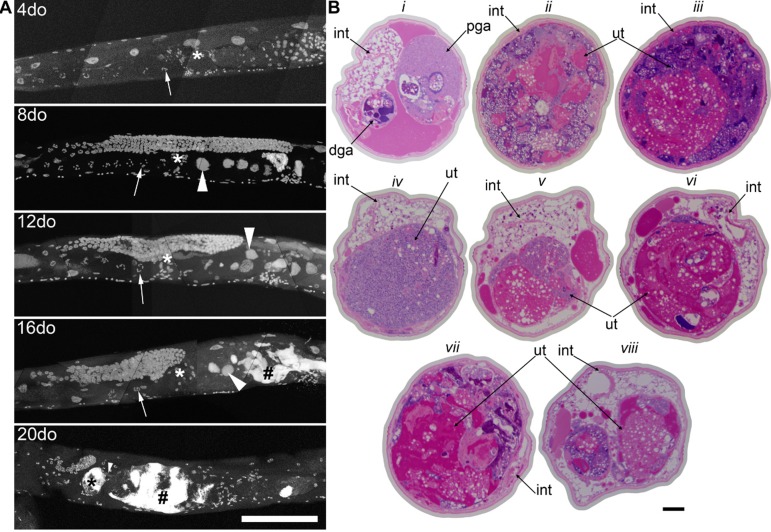
Germline masses accumulate with age **a,** Partial maximum projection of mid-section of whole wild-type worms stained with DAPI at 4, 8, 12, 16, and 20-days-old raised at 20 degrees. Arrow indicates the -1 oocyte in diakinesis (none visible in 20-day-old worm). Asterisk indicates spermatheca. Large arrowhead indicates unfertilized, endomitotic nuclei. # indicates a mass that has no distinct cellular structure. Small arrowhead in the 20-day-old indicates site of invasion from uterus to spermatheca. Scale bar represents 100 microns. **b,** Cross sections of a 20-day-old wild-type worm stained with pararosaniline and methylene blue. Cross sections from the same worm starting at approximately -1 oocyte position along anterior half (*i*). Remaining sections are spaced 50 μm apart (*ii-viii*), moving towards posterior and ending at approximately -1 oocyte position along posterior half (*viii*). In some cross sections, the uterine growth has taken up nearly the whole diameter of the worm (*ii*, *iii*, *vii*). Growth recedes at midbody (*iv*). Intestine (int), distal gonad arm (dga), proximal gonad arm (pga), and uterus (ut) are indicated.

**Figure 2:**
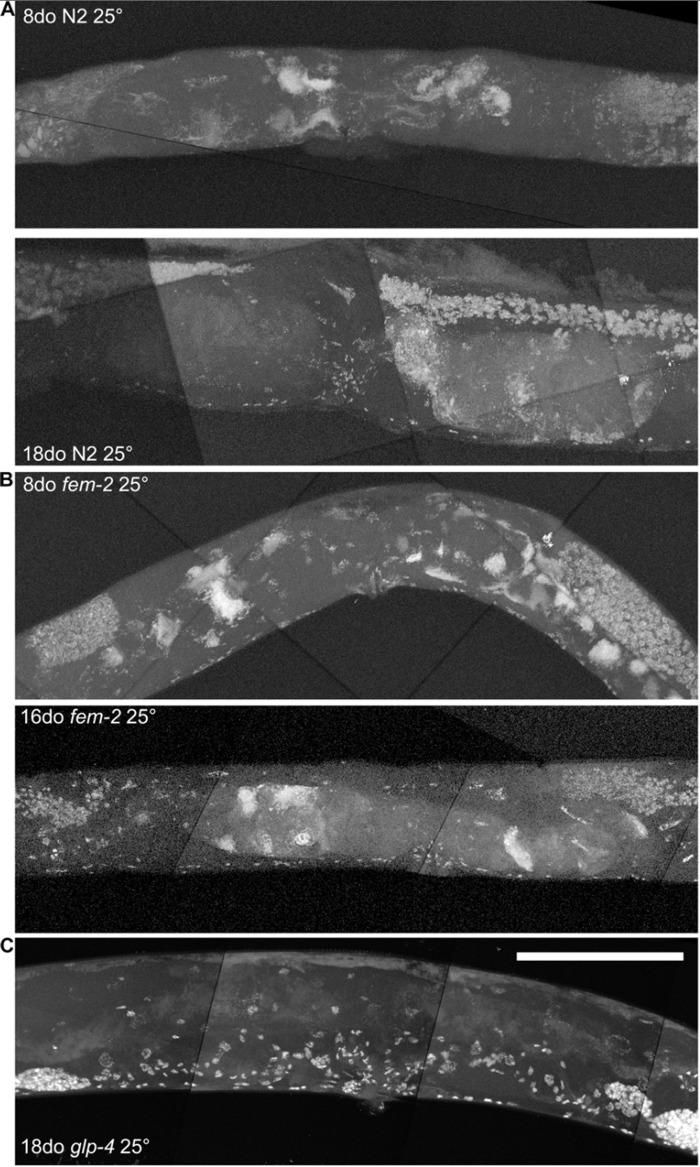
Age-related growths require a germline, but not competent sperm **a,** Maximum projection of 8 and 18-day-old wild-type worm raised at 25 degrees and stained with DAPI. Growths visible at both ages, though generally never as severe as old wild-type worms raised at 20 degrees. **b,** Maximum projection of 8 and 16-day-old *fem-2* (no sperm) worms raised at 25 degree restrictive temperature and stained with DAPI. Masses are visible at both ages, similar to wild-type worms. **c**, Maximum projection of 18-day-old *glp-4* (underdeveloped gonad and germline) worm raised at 25 degree restrictive temperature and stained with DAPI. No masses are visible. Scale bar represents 100 microns.

**Figure 3:**
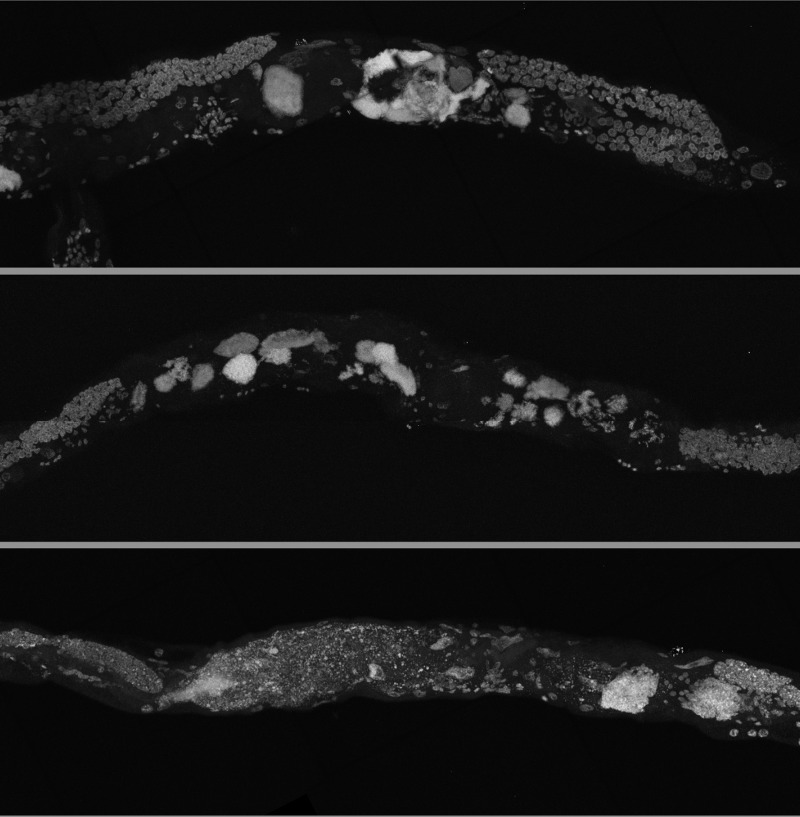
Germline masses in worms grown in axenic media Partial maximum projection of mid-section of 26-day-old whole wild-type worms stained with DAPI. Worms were raised at 20 degrees in axenic media.

By using a combination of novel imaging techniques (Figure 1, Movies 1-5), we have refined the spatial position of the midbody DNA masses we previously reported [16] primarily to the uterus, through virtue of being able to analyze the animal in detail in 3-dimensions. The masses begin to grow as early as 8 days of age (Figures 1A-B, Movie 1). This is consistent with the approximate age *C. elegans* deplete their sperm [10,26] and oocyte quality declines such that fertility can only be extended a few days by mating [1,11,12]. Once oocytes fail to be fertilized in the spermatheca, they bypass diakinesis arrest [6] and begin endoreduplicating to swell the uterus by 16 days of age (Figures 1A, Movie S1). The DNA masses are sometimes pushed into the continuous spermatheca and proximal gonad arm from the uterus (Figure 1A), similar to tumors that arise in young adult *glp-1* mutants [27,28]. Large age-related uterine growths can sometimes rupture regions of the uterine wall, causing them to enter the pseudocoelom(data not shown). Aged wild-type worms raised at elevated temperature have generally less severe uterine masses by DAPI staining than those raised at normal temperatures (Figure 2), but the masses also generally become visible at earlier ages, compared to worms raised at standard temperature (20°C). Further, these small masses are more often seen in the distal and proximal gonad arm rather than in the uterus (data not shown). We also observed uterine growths by DAPI staining in aged animals grown in sterile conditions, using axenic culture methods (Figure 3). Therefore, the growth of these uterine masses is not caused solely by bacterial infection. The reduced severity of uterine masses does not mean bacteria contribute to growth of uterine masses, since axenic growth conditions also cause slow growth. We observed no masses, even at advanced ages, in *glp-4* mutants that do not develop a full gonad (Figure 2C). We did observe germline masses in old, sperm-deficient *fem-2* worms similar to old wild-type worms (Figure 2A-B), and we previously demonstrated that no masses form in males [16]. This is also consistent with previous studies that reported an Emo phenotype in young worms devoid of sperm [10,17].

### Germline masses are responsible for an age-related genome copy number increase

In order to quantify the genomic DNA copy number of these growing uterine masses in aging animals, we used digital PCR of individual nematodes to estimate genome copy number at different ages. In a previous study by us, we used qPCR to estimate genome copy number in select strains [16]. However, here instead of qPCR, we use the digital PCR technique, as it is very sensitive, highly resistant to PCR inhibitors, and allows absolute quantitation of low copy number without reliance on standards [29]. The expected genome copy number of an adult hermaphrodite is ~5434 (3134 somatic) (Table 1), not including fertilized embryos.

**Table 1:** Expected genome copy number of a wild-type adult hermaphrodite

	Germline Nuclei	Somatic Nuclei	Intestine	Hypodermis	Sperm	Embryos	TOTAL (no embryos)	TOTAL (6 embryos)
# Nuclei	1000	959	34	98	150	520		
Number	2	1	1	1	2	6		
Ploidy	1	2	32	4	1	2		
								
Total	2000	1918	1020	196	300	6240	5434	11674

The number of embryos, which can be as many as 25-30 in the most extreme cases [26], and developmental stage of embryos in the uterus can vary greatly, causing a high degree of variability in the measured genome copy number of reproductive adults. To quantitate the amount of individual variation that is also visually apparent by microscopy (Movies 3-5), we assayed individual animals rather than pools of animals, giving us insight into the variance of age-related genome copy number variation between individual worms. We calculated a mean genome copy number of 17,160 (n = 10, +/− 3,102 S.E.M.) at 3 days of age (Table 2, Figure 4). Genome copy number does not significantly change from 3 days to 12 days of age, but there is almost a 4-fold increase in genome copy number between 12 and 16 days of age (p<0.0001, Figure 4). It is worth noting that this 4-fold increase in genome copy number is substantially increased over that of younger animals, when reproduction is at peak capacity, and the animals are packed with developing oocytes and embryos. The trends we report here of genome copy number increases with age using digital PCR agree with our previous study [16] and also correlates with the onset of uterine growths (Figure 1A). *glp-4* mutants lack a gonad and have much lower genome copy number and reduced variance compared to wild-type worms (Figure 4). Average genome copy number in *glp-4* worms decreases between days 6 (6345, n = 10, +/− 312 S.E.M.) and 9 (3781, n = 9, +/− 718 S.E.M., p=0.0034) and then remains relatively constant through day 16. We previously were unable to calculate genome copy number in this strain by qPCR [16] likely due to overall low copy number or potential PCR inhibitors in preparing nucleic acids from individual worms. This data suggests the high variance in wild-type genome copy number is primarily due to germline effects. Initially the variance in genome copy number in young worms is likely due to developing embryos, while as the animal ages, the substantial variance increase arises due to massive proliferation of unfertilized, endore-duplicating oocytes, an age-related Emo phenotype.

**Table 2:** Measured genome copy number by digital PCR

Age (days)	N2	*glp-4*	*cep-1*	*daf-2*
3	17160 +/− 3102 (n=10)	5735 +/− 653 (n=10)	11644 +/− 1672 (n=9) (1 low)	-
6	24041 +/− 4333 (n=10)	6345 +/− 312 (n=10)	26337 +/− 3383 (n=10)	-
8	-	-	-	51068 +/− 8123 (n=10)
9	18130 +/− 3129 (n=10)	3781 +/− 718 (n=9)	33210 +/− 4674 (n=9)#	-
12	15360 +/− 2497 (n=10)	4054 +/− 404 (n=9)	45600 +/− 9960 (n=10)	-
16	60139 +/− 7004 (n=10)	3979 +/− 540 (n=9)	53411 +/− 13202 (n=8)&&	-
32	-	-	-	16609 +/− 2172 (n=10)
45	-	-	-	9427 +/− 1707 (n=10)
60	-	-	-	24086 +/− 7849 (n=10)

Error is S.E.M.

& Indicates censored animal that was below detection threshold

# Indicates censored animal that was above detection threshold

**Figure 4:**
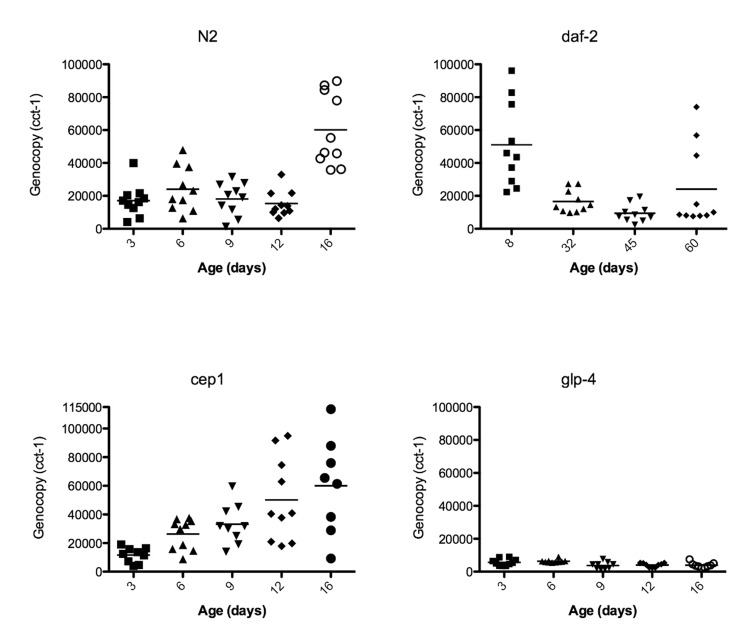
Genome copy number increases with the growth of uterine masses Data points represent the genome copy number (y-axis) of individual worms at different ages (x-axis) as measured by digital PCR of individual worms with primers corresponding to genomic DNA of *cct-1* gene. Graphs are of wild-type N2, *glp-4*, *daf-2*, or *cep-1* worms.

### Age-related germline masses have a complex composition

**Figure 5:**
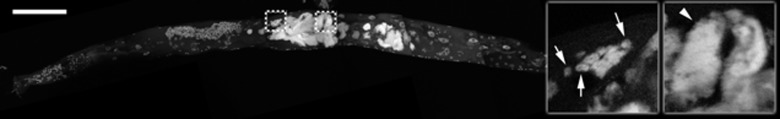
Uterine growths contain individual nuclei and large masses of chromatin Partial maximum projection of a 16-day-old wild-type animal stained with DAPI and raised at 20 degrees. Insets are a single focal planes enlarged from the boxed regions. Arrows point to some of the visible individual nuclei in the uterus. Arrowhead points to a large chromatin mass in the uterus. Scale bar represents 100 microns.

Examination of the germline masses by staining of sectioned tissues and imaging by light microscopy (Figures 1B, Movies 3-5) reveals three regions with distinct characteristics: cellularized areas with similar appearance to primary oocytes, chromatin masses associated with abnormal nuclei, and acellular accumulations in extracellular space of yolk and/or other matter. While the morphology of aged worms varies greatly, we found that 20-day-old animals have very large uterine masses that compress many tissues. These masses appear to be smallest near the vulva, suggesting that the local microenvironment inhibits growth. This could be due to several causes, including extracellular signaling, or bacterial invasion through the vulva [30]. However, as masses are still present in worms grown in axenic media, this is unlikely. We found that while some nuclei in the uterine masses appear intact, other regions appear to be primarily acellular accumulations of chromatin and yolk based on histological staining. We also observed clusters of individual nuclei and large, endoreduplicating nuclei in these masses by DAPI staining (Figure 5). Defects in nuclear envelope morphology, such as blebbing, are a known age-related degenerative process in *C. elegans[31]* and could play a role in the growth of these masses. Lamin appears to surround the periphery of some, but not all, of these masses by antibody staining and lamin::gfp reporters (data not shown). Indeed, it is difficult to delineate individual cellular boundaries in these masses, suggesting that DNA replication is occurring within the uterus unbound by cell membranes.

**Figure 6:**
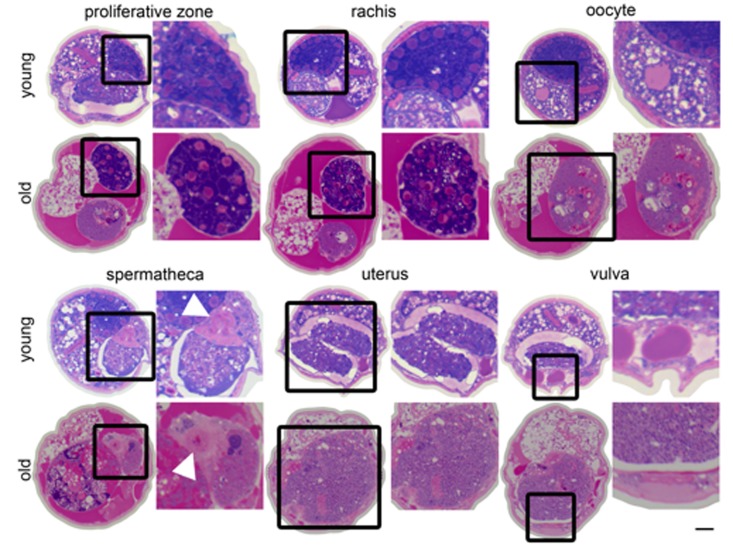
Changes in the germline with age Cross sections from a 4-day-old (young) and a 20-day-old (old) wild-type worm. Regions of the germline indicated above the sections are representative and the structure is enlarged to the right of each section. White arrowhead indicates spermathecal epithelium. The region of the uterus shown in the old animal is near the vulva does not contain a massive uterine growth.

Unfertilized oocytes in the proximal gonad arm of old animals are also clearly abnormal compared to those in young animals. Young healthy oocytes are large with distinct nuclei, while old animals often have smaller oocytes with less distinct nuclei and are filled with other unidentified matter not visible in young oocytes (Figure 6, Movies 2-5). The technique for visualizing individual animal morphology we have employed here in animals across the full lifespan of *C. elegans* allows a dynamic visual appreciation of the development of low quality oocytes, compared to more traditional imaging techniques and is in agreement with previous reports of smaller oocytes that are incapable of normal embryonic development in middle aged animals [11,12].

A large portion of the non-cellularized swelling of the midbody appears to be composed of yolk deposited in the pseudocoelom, which is magenta-colored in the stained cross sections (Figures 1B, 6). In young worms, yolk is produced in the intestine and transported through the pseudocoelom to the germline [32,33]. Although some yolk is always present in the pseudocoelom of adult worms, there is an accumulation of yolk outside the intestine with age, when the somatic gonad no longer holds enough mature oocytes to capture this material by endocytosis [30,32,34,35].

In addition to the uterine mass phenotype, the distal gonad is highly disorganized and shrunken in old animals compared to young. Instead of meiotic germline nuclei surrounding the periphery of the gonad with a well-developed central rachis as in young worms [22], nuclei appear to be spread throughout the gonad often with no visible rachis (Figure 6, Movies 2-5). These nuclei also appear to be generally less regular in size and composition. Twenty-day-old animals also have a shrunken distal gonad that is also visible by DAPI staining (Figure 1A).

### The *daf-2* insulin-signaling mutant is able to reduce uterine masses at extreme age

**Figure 7:**
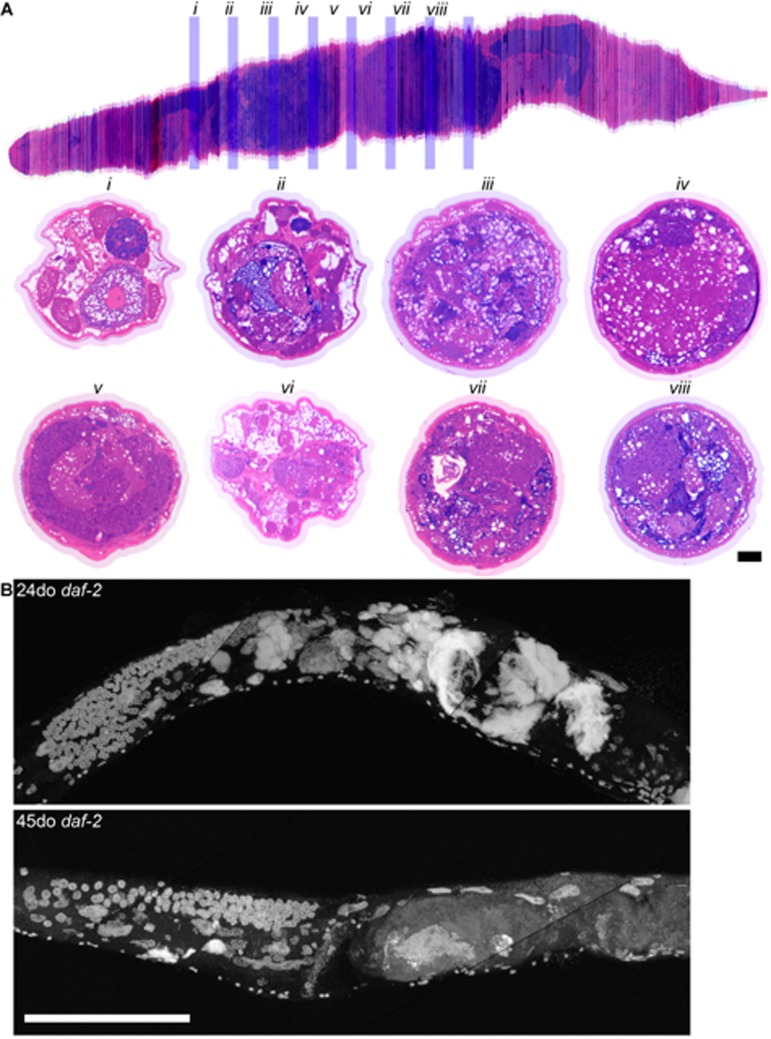
Long-lived *daf-2* mutants have decreased uterine masses with age **a,** Aligned cross sections of a 20-day-old wild-type worm stained with pararosaniline and methylene blue. The aligned cross sections were resliced longitudinally in software (top) from the mid-pharynx (top left) to the tail (top right). Cross sections from the same worm containing the gonad are shown spaced 50 μm apart *(i-viii*) and are represented by blue bars in the longitudinal section (top). In some cross sections, the uterus has enlarged to take up nearly the whole diameter of the worm *(iii*, *iv*, *vii, viii*). Intestine (int), distal gonad arm (dga), proximal gonad arm (pga), and uterus (ut) are indicated. **b,** Maximum projection of 24 and 45-day-old long-lived *daf-2* worms raised at 20 degrees and stained with DAPI. Large uterine masses, similar to those seen in old wild-type worms, are visible at 24-days-old, but are reduced in size at 45-days-old. Scale bar represents 100 microns.

Because long-lived *daf-2* worms are resistant to *gld-1* driven tumors in young animals [18] and we have previously seen lower genome copy number in old *daf-2* worms compared to wild-type [16], we evaluated in greater detail whether age-related uterine growths were delayed in *daf-2* animals. The increased resolution of imaging surprisingly showed that at 20 days of age, *daf-2* germline masses (Figures 7, Movie 6) are visually very similar to wild-type (Figure 1, Movies 3-5). These masses become less severe with age by both DAPI staining and genome copy number measurements after peaking in severity around 20 days of age (Figures 4, 7, Table 2). Fourty-five-day-old *daf-2* worms, well past the maximum life span of wild-type worms, have barely visible uterine masses by DAPI staining (Figure 7), and lower genome copy number measurements (Figure 4, Table 2). The uterus still appears swollen in 45-day-old animals even though DAPI intensity is drastically lowered (Figure 7), suggesting there could be a reduction of DNA in these masses but not an actual decrease in size. Eight-day-old *daf-2* worms have a strikingly elevated genome copy number(51,068, n = 10, +/− 8,123 S.E.M.) compared to 9-day-old wild-type animals, representing an almost 3-fold increase in genome copy number over wild-type levels, followed by a significant decrease at 32 (16,610, n = 10 +/− 2172 S.E.M., p=0.0007) and 45 (9,427 +/− 1,707 S.E.M., *p*=0.02) days of age. At 60 days of age the variance of genome copy number increases (*p* = 0.0001), and there may be some reoccurrence of endoreduplication at the end of life in the *daf-2* longevity mutant. Average genome copy number increases slightly, but not significantly between 45 and 60 days of age (24,090, n = 10, +/− 7,849 S.E.M., p=0.08). The substantial increase in genome copy number in *daf-2* animals at a young age is particularly unexpected, as it is known that this strain of *daf-2* is less fertile than N2 [36]. The origin of these extra genome copies in *daf-2* animals warrants further investigation, as it is either due to reduced oocyte quality, resulting in an early endomitotic phenotype not detectable by our methodology (DAPI staining or serial sectioning/3D reconstruction), or is somatic in origin.

### *cep-1*/p53 has earlier onset of endoreduplicating uterine growths, and *cep-1*/p53 declines with age

**Figure 8:**
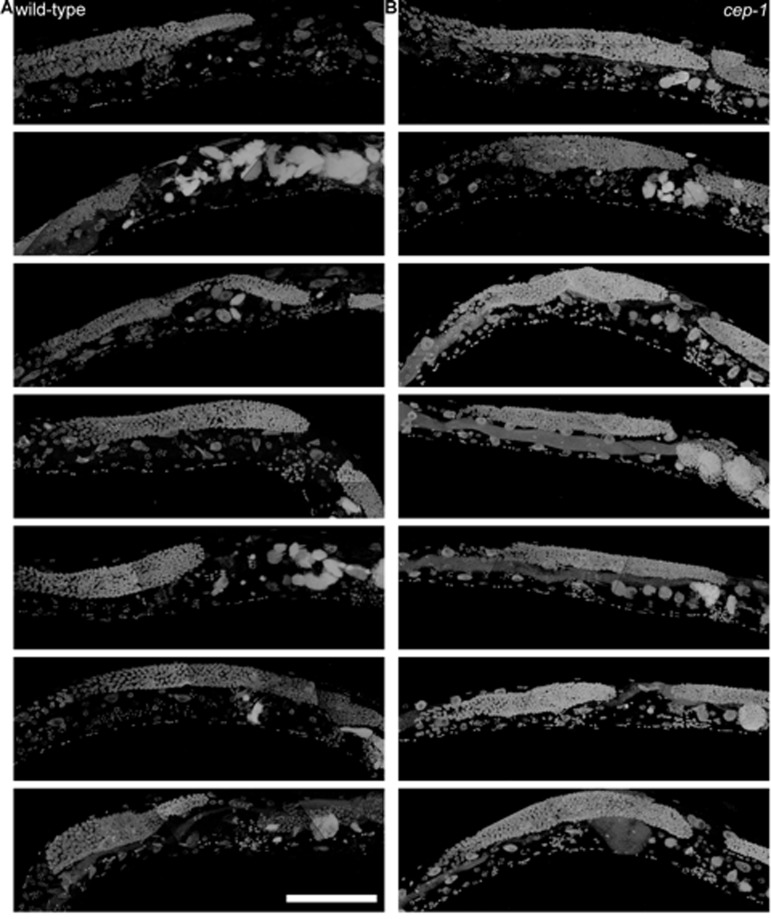
8-day-old wild-type and *cep-1* worms have visibly detectable masses **a,** Partial maximum projection of mid-section of 7 individual whole 8-day-old wild-type worms stained with DAPI raised at 20 degrees. **b,** Partial maximum projection of mid-section of 7 individual whole 8-day-old *cep-1* worms stained with DAPI raised at 20 degrees. Uterine masses are detectable at the same age in *cep-1***(b)** and wild-type **(a)**. Scale bar represents 100 microns.

**Figure 9:**
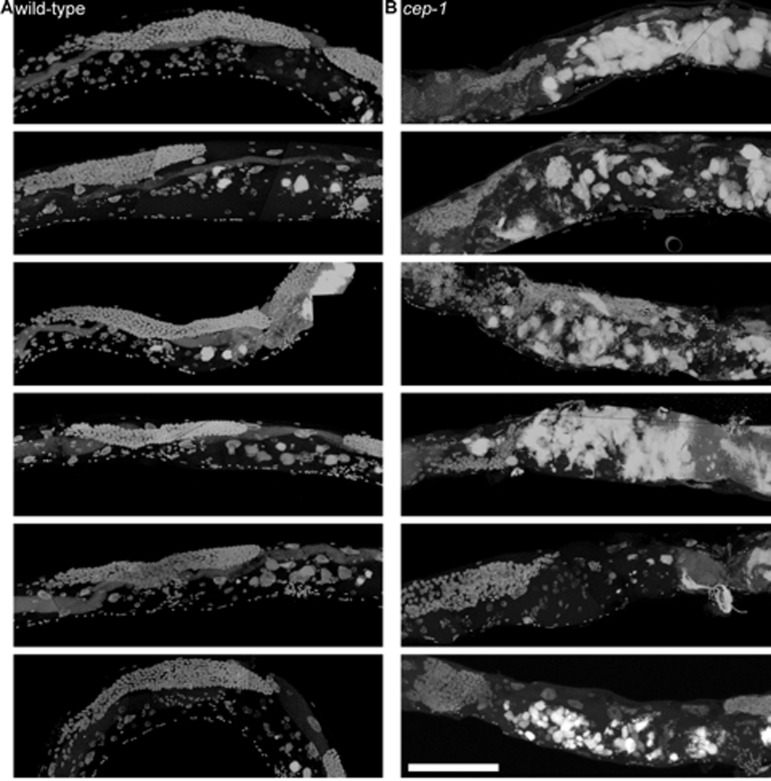
*cep-1* worms have earlier uterine mass onset **a,** Partial maximum projection of mid-section of 6 individual whole wild-type worms stained with DAPI 12-days-old raised at 20 degrees. **b,** Partial maximum projection of mid-section of 6 individual whole cep-1 worms stained with DAPI 12-days-old raised at 20 degrees. Uterine masses are visibly larger in cep-1 **(b)** than wild-type **(a)**. Scale bar represents 100 microns.

**Figure 10:**
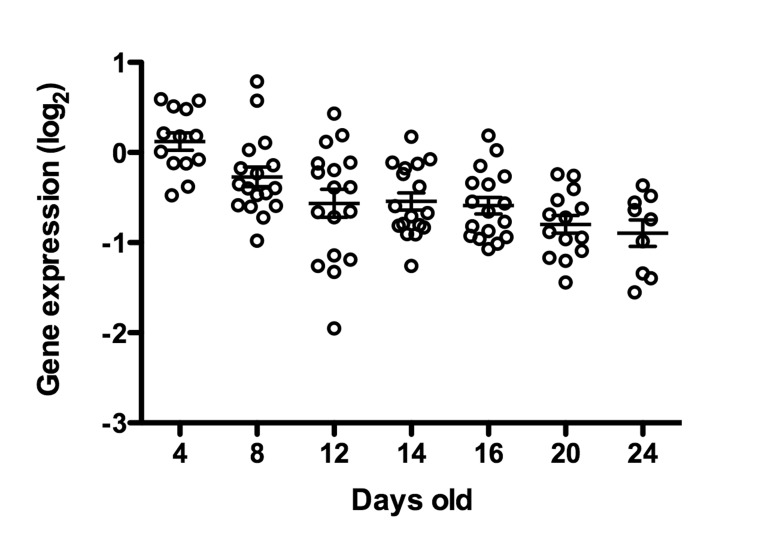
*cep-1*/p53 transcript abundance decreases with age Whole genome expression profiling was used to identify genes that were differentially expressed with age (for complete description, see [19], cep-1/p53 is statistically significantly differentially expressed with age relative to young worms (p<0.05, *cep-1*/p53 is in Cluster 5 [19]). Each data point represents the *cep-1*/p53 message levels in a single animal, and expression level is represented relative to mRNA from a pool of young N2 animals [19].

**Figure 11:**
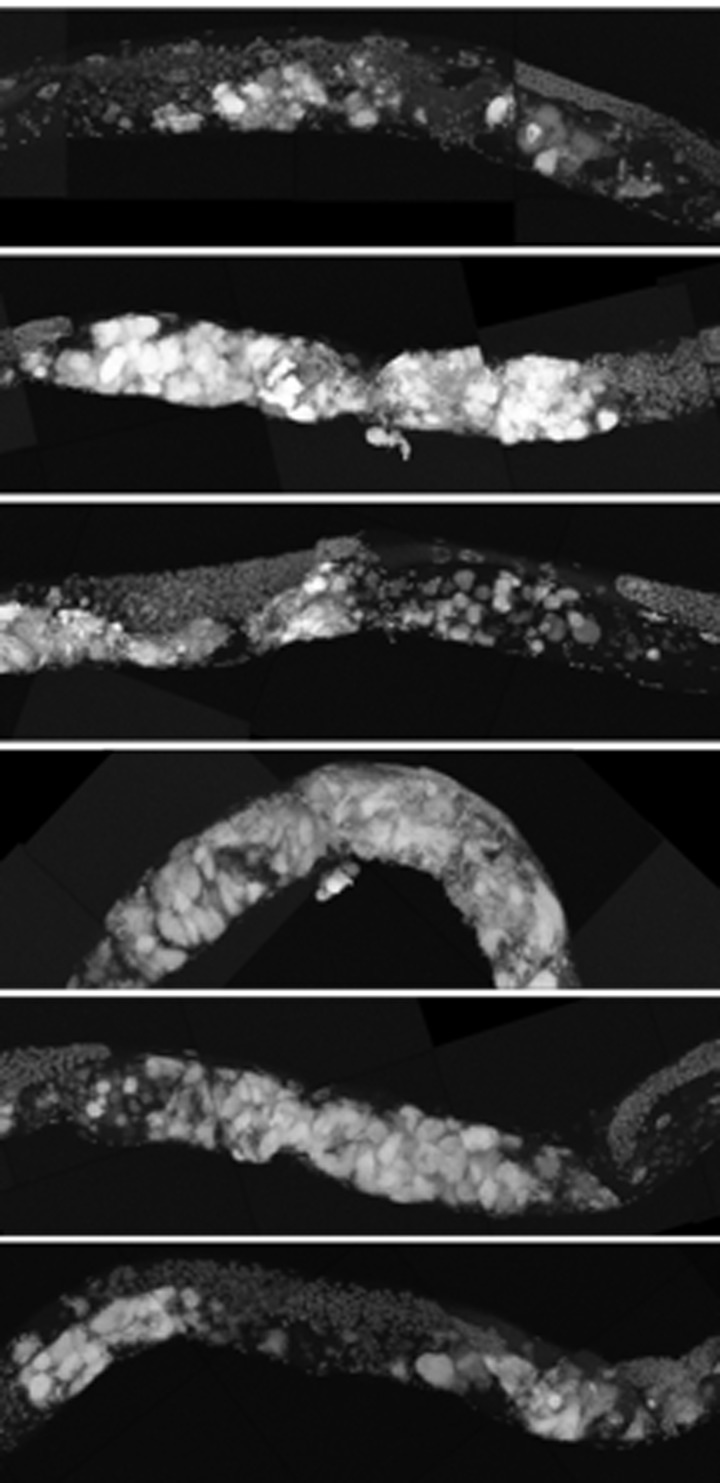
*ced-3* animals have early uterine mass onset Partial maximum projection of mid-section of 6 individual 12-day-old *ced-3* worms stained with DAPI and raised at 20 degrees. 12-day-old *ced-3* animals have uterine masses visually similar to *cep-1* at the same age.

Because *C. elegans cep-1* is known to function in DNA damage-induced apoptosis in the germline [37-39] and the mammalian homolog p53 is a well-known tumor-suppressor [40,41], we tested the hypothesis that *cep-1* limits the growth of these age-related uterine masses either through it's role in germline apoptosis or in transcriptional regulation of other genes. Indeed, we observed large chromatin masses in the uterus of most *cep-1* worms by middle age (12 days of age, Figures 8-9), whereas most wild-type worms do not have similar growths until about 16 days of age (Figure 1A, Movie 1. The age at which these masses can be morphologically visualized in cep-1 mutants is roughly equivalent to when they appear in wild-type (Figure 8). Mating *cep-1* hermaphrodites to wild-type males did not appear to delay onset (data not shown), suggesting oocyte defects rather than unviable sperm causes endoreduplication as in middle aged wild-type worms [1,11]. *Cep-1* worms have a steady increase in genomic DNA copy number from 3 to 16 days of age (Figure 4, Table 1), whereas wild-type worms maintain relatively steady levels of genomic DNA before a large increase at 16 days of age (Figures 4, Table 1). *Cep-1* worms have a significantly higher genome copy number than wild-type worms at 9 (p = 0.01) and 12 (p = 0.002) days of age. Given wild-type animals accumulate uterine masses with age, and loss of *cep*-*1* increases the rate at which these masses appear, we wished to evaluate the transcriptional levels of *cep-1* with age to determine if there was any change in *cep-1* with increasing age. We had previously reported the largest study on gene expression profiling and aging in any species to date in *C. elegans [19]*, so we mined this pre-existing data set to look at the transcriptional profile of *cep-1* in wild-type aging of *C. elegans*. In our previous study, we noted that there were 5986 genes that were statistically significantly differentially expressed over the lifetime of *C. elegans*. These genes clustered into seven distinct profiles over lifespan, and *cep-1* is in cluster 5 (supplemental Table 8, [19]), and falls steadily in abundance over the life of *C. elegans* from a peak in young adulthood (Figure 10). Hence, there is a statistically significant decline in the transcript abundance of a key mediator of DNA damage and cell growth with age in *C. elegans*. To test whether DNA damage-induced apoptosis is important in removing oocytes likely to become uterine masses, we also examined *ced-3* mutants for uterine masses, which are incapable of physiological or DNA damage-induced apoptosis [42]. In agreement with previous studies that found loss of *ced-3* results in small, low-quality oocytes that will undergo endomitosis [14,15,43]. We observed an early onset of massive uterine growths in *ced-3* worms (Figure 11). This indicates that DNA damage may be occurring in these uterine growths and DNA damage-induced apoptosis may be a contributing mechanism to delay these massive uterine growths.

## DISCUSSION

We have described in detail, a number of novel phenotypes in the aging *C. elegans* germline. Although age-related endoreduplication of unfertilized oocytes in middle aged animals has been commented on previously [1,11,16], we used a number of new techniques to describe the pathology of these masses and other age-related germline phenotypes in greater detail, and for the first time in older animals on different genetic backgrounds that may help shed light on the mechanism of age-related germline phenotypes in future studies. Most striking is the massive growth in the uterus that arises from unfertilized oocytes. These masses grow large enough to swell the uterus to fill most of the diameter of the worm. They are primarily composed of endoreduplicating unfertilized oocytes, masses of chromatin from presumably lysed nuclei, small clusters of individuals cells and/or nuclei, and yolk proteins. The growth of these uterine masses is accelerated in the *cep-1*/p53 mutant known to be required for DNA damage-induced apoptosis in *C. elegans*.

*Cep-1* is not a close homolog of mammalian p53 and was not identified as such until 2001 [20,21]. Although we show that loss of *cep-1* causes early age-related uterine masses, a previous study showed that *cep-1* does not affect the number of mitotic cells in germline tumor mutants or the shortened lifespan of these mutants [18]. Therefore it is likely that *cep-1*/p53 has a different mechanistic function in *C. elegans* tumors from *gld-1* and *glp-1* [28,44] mutants compared to mammals [45]. *Cep-1*/p53 controls the transcription of many downstream target genes and affects processes such as DNA damage-induced apoptosis [20,21], meiotic segregation in the germline [20], stress tolerance [46], genotoxic stress response [24], and cell growth [24,43]. We observed a statistically significant decrease in the transcriptional abundance of *cep-1*/p53 with age, concordant with an increase in endoreduplication within the gonad, contributing to late life pathology of the gonad. The reason for the decrease in *cep-1*/p53 with age within *C. elegans* is currently not known. However, in mammals, p53 activity also declines with age, and this decrease has been proposed to be a contributing factor to the increased tumor incidence in older populations [41], as well as being linked to cellular senescence [47], and antagonistic pleiotropy in aging [48]. Intriguing links between the activity of p53, mTOR, and senescence [49,50], hint at a more complex explanation for the increase in the Emo phenotype in late life than a simple decrease in apoptosis due to the decline in levels of *cep-1*/p53 with age. One possible explanation for the Emo phenotype in the aged *C. elegans* gonad is the gradual decline in *cep-1*/p53 activity results in reduced apoptosis, combined with enhanced activity of the mTOR growth program, which then gives rise to the Emo phenotype with age. This decline in *cep-1*/p53 in aging *C. elegans*, coupled with a rapid increase in growth in the gonad (as evidenced by an upto 4 fold increase in overall genome copy number per worm) hints at possible commonalities in widely divergent species in age-related phenotypes due to the decline of p53 levels with age.

It also appears likely that DNA damage-induced apoptotic defects cause early uterine growths based on our observations and those of others [14,15,43]. The massive increase in DNA with age, in conjunction with decreased levels of *cep-1*/p53, coupled with clear pathological outcomes (compression of a variety of tissues with likely functional consequences) begs the question whether or not this age-related pathology could be considered to be analogous to age-related tumors seen in mammalian systems. By classical criteria, there are almost no tumors in *C. elegans* described to date that conform to a formal definition of cancer. The closest exception to this generalization may be the laminin mutants previously reported [51], where germ cells were documented to be actively invading somatic tissues, in conjunction with midbody swelling. What would be needed to legitimately link the age-related pathology we describe here in *C. elegans* to cancer are additional criteria, such as mutations in tumor suppressors with age that are then intimately linked to the development of the tumor-like pathology. The age-related germline dysplasia we describe here shares several characteristics with those observed in tumor-prone mutants such as *gld-1*, but also have several distinctions. They are both abrogated in*daf-2* worms for a time and are the result of uncontrolled germline growth. However, *daf-2* worms do not have a delayed age-related germline tumor onset as seen in the tumor-prone mutants.

Instead, it is likely there must be a mechanism to reduce tumors at extreme ages after they have already formed. Also distinct from age-related tumors, *gld-1* and*glp-1* tumors appear to have more rapid growth of intact germline nuclei while the age-related dysplastic pathology in the gonad grows more slowly and contains regions that are both cellularized and acellular, as well as accumulations of material such as yolk and chromatin.

The molecular characterization of this unique age-related gonadal pathology in *C. elegans* could potentially give more insight into age-related tumors in other species. Of particular interest will be whether age-related mutations in orthologs of mammalian tumor suppressors or oncogenes could be driving the growth of this dysplastic gonadal pathology, as we showed is possible in the *cep-1/*p53 mutant. Indeed, the rapid increase in genomic DNA at extreme age could be a source of genomic DNA changes and there are established methods to evaluate genomic DNA quality in *C. elegans* [52,53].

We propose that age-related uterine masses arise from a combination of several different events. Some regions of the uterine masses appear cellularized while others are acellular. Oocytes that normally arrest at diakinesis eventually bypass this arrest and begin endo-reduplicating. The nuclear envelope breaks down as the amount of DNA increases, causing the cell to eventually lyse. Many of these nuclei have a disrupted nuclear envelope as observed by lamin::gfp (data not shown). Degradation of the nuclear envelope has been observed in aging *C. elegans* previously [31]. As oocytes begin to be ovulated at a slower rate in the aging mother, yolk is no longer being used at the same rate by primary oocytes, which would account for the buildup of yolk in the pseudocoelom and masses we and others have observed [30,33-35]. The intestine is apparently unable to regulate the amount of yolk produced to match actual demand [35]. This is possibly also influenced by the loss of intestinal nuclei which occurs as *C. elegans* ages, which we recently described [30].

We have demonstrated that several age-related changes occur in the *C. elegans* gonad. Most dramatically, we describe how uterine masses in *C. elegans* arise in the aging germline from unfertilized oocytes that begin endoreduplicating after bypassing the arrest at the end of diakinesis [5]. Uterine masses grow uncontrollably until they occupy most of the diameter of the worm and are accompanied by a massive increase in DNA copy number per worm in old adults, which greatly exceeds the genome copy number of worms at their reproductive peak at younger ages, and is coupled to a decline in levels of *cep-1*/p53. We also determined that loss of the CEP-1/p53 protein exacerbates the growth of uterine masses at earlier ages. DNA damage-induced apoptosis likely limits uterine growths, as *cep-1*/p53 is required for DNA damage-induced apoptosis in the germline [54]. Additionally, we and others have found that *ced-3* lowers oocyte quality and enhances the endomitotic phenotype. We have also found that the occurrence of these masses is not completely dependent on the bacterial food source or immune response [55], as worms grown in sterile axenic media still develop uterine masses with age. In summary, the aging *C. elegans* uterus develops massive growths from endoreduplicating oocytes that are modulated by the CEP-1/p53 protein, which has multiple functions including roles in DNA damage response, growth [41,45,48], genotoxic stress response [24], and cellular senescence [47].

## EXPERIMENTAL PROCEDURES

*C. elegans* strains were cultured using standard conditions [22] (N2, *cep-1(gk138)I*, *daf-2(e1370)III, ced-3(n717)IV)* or at the restrictive temperature of 25 degrees (*glp-4(bn2)I*,*fem-2(b245)III*) and obtained from the *Caenorhabditis* Genetics Center. Synchronized populations were acquired by allowing adult parents to lay eggs for 2-4 h before removing them (day 0). Worms were transferred daily to fresh plates during the reproductive period. Confocal microscopy, staining of tissue sections, and 3-D visualization was performed as described [30].

Individual worms for genome copy number experiments were frozen in water and lysed in Proteinase K as described [16]. Digital PCR was performed on the lysate of individual animals using primers corresponding to the genomic DNA of the *cct-1* gene (5'-aatacggttgtgtttcaggtaatg-3' and 5'-taccggtgagggcaa gaat-3'). Digital PCR of *ced-7* and *fat-3* genes yielded similar results to *cct-1* (data not shown). Digital PCR signal was measured using UPL probe #78 (Roche) on a Biomark microfluidic system in 12.765 digital arrays (Fluidigm).

For Axenic liquid culture, *C. elegans* were first grown at 20C at a density of 15,000 worms/plate on NGM plates seeded with OP50 bacteria. Eggs were isolated from these cultures and grown in CeHR medium according to previous studies [56,57] with a minor modification. Heme was excluded from the axenic media and was added prior to use along with the UHT milk. Ten freshly hatched sterile L1 were seeded in 1 ml CeHR medium without antibiotics in a 24 well plate and grown at 20C shaking at 450 rpm on a small shaker. After the first generation, gravid adults were allowed to lay eggs for 4 hours in each well and were subsequently removed. Synchronized eggs were allowed to grow in sterile CeHR medium until Day 22 of adulthood. The media was changed three times weekly until day 8 of adulthood, and from there it was changed twice weekly. The first generation of nematodes grows slowly on CeHR [7-10] days, while successive generations grow at similar rates to those on solid media. Nematodes were allowed to adapt to CeHR media for at least one full generation before use.

## MOVIES

Movie 1 This movie shows a 3-D model of DAPI staining in a 4-day-old worm, with germline nuclei colored red. The non-germline nuclei disappear, showing only the germline. Representative 8, 12, and 16-day-old germlines are then shown, with a massive endoreduplicating uterine mass visible by day 16.

Movie 2 This movie shows all the aligned tissue sections of a single 4-day-old wild-type worm (w2).

Movie 3 This movie shows all the aligned tissue sections of a single 20-day-old wild-type worm (w2).

Movie 4 This movie shows all the aligned tissue sections of a single 20-day-old wild-type worm (w3).

Movie 5 This movie shows all the aligned tissue sections of a single 20-day-old wild-type worm (w4).

Movie 6 This movie shows all the aligned tissue sections of a single 20-day-old daf-2 worm (w1).
